# Influence of Ambient Temperature on Radiative and Convective Heat Dissipation Ratio in Polymer Heat Sinks

**DOI:** 10.3390/polym13142286

**Published:** 2021-07-12

**Authors:** Jan Kominek, Martin Zachar, Michal Guzej, Erik Bartuli, Petr Kotrbacek

**Affiliations:** Heat Transfer and Fluid Flow Laboratory, Faculty of Mechanical Engineering, Brno University of Technology (BUT), Technicka 2896, 616 69 Brno, Czech Republic; Martin.Zachar@vut.cz (M.Z.); Michal.Guzej@vut.cz (M.G.); Erik.Bartuli1@vut.cz (E.B.); Petr.Kotrbacek@vut.cz (P.K.)

**Keywords:** polymer heat sink, thermal management, thermal conductivity, radiation, convection, composites

## Abstract

Miniaturization of electronic devices leads to new heat dissipation challenges and traditional cooling methods need to be replaced by new better ones. Polymer heat sinks may, thanks to their unique properties, replace standardly used heat sink materials in certain applications, especially in applications with high ambient temperature. Polymers natively dispose of high surface emissivity in comparison with glossy metals. This high emissivity allows a larger amount of heat to be dissipated to the ambient with the fourth power of its absolute surface temperature. This paper shows the change in radiative and convective heat transfer from polymer heat sinks used in different ambient temperatures. Furthermore, the observed polymer heat sinks have differently oriented graphite filler caused by their molding process differences, therefore their thermal conductivity anisotropies and overall cooling efficiencies also differ. Furthermore, it is also shown that a high radiative heat transfer leads to minimizing these cooling efficiency differences between these polymer heat sinks of the same geometry. The measurements were conducted at HEATLAB, Brno University of Technology.

## 1. Introduction

With new advancements in electronics, especially in their miniaturization, new cooling challenges need to be met so that the electronics’ efficiency and service life would not decrease. Polymers, due to their easy fabrication, low cost and low weight, are already finding their ways in many active cooling applications despite their low thermal conductivity as polymer heat exchangers in cooling electronics and also in the automotive industry [1,2,3,4]. Their amorphous structure responsible for their low thermal conductivity prevents their usage in passive cooling applications as heat sinks. This disadvantage could be eliminated if the right fillers with high thermal conductivity would be incorporated into the base polymer matrix using modern production techniques, these composites could be used in electronics passive cooling applications where they would bring with them other polymer benefits [5,6,7,8,9,10,11]. High thermally conductive polymers could be used as heat sinks especially in applications where high ambient temperature is expected.

Modern polymer blends offer high thermal conductivity, but in comparison with traditionally used aluminum and copper, their conductivity still remains low approximately by tenfold. However, they could be used in applications where the heat transfer via conduction inside the material is not restricting the overall heat transfer from the cooled part to the ambient, which is typical in passive cooling using a heat sink [4]. Especially in applications where heat transfer from the heat sink by convection is strictly limited (enclosed space) or where their naturally high emissivity would lead to high heat transfer via radiation (applications with high ambient temperature).

Behavior of polymer heat sinks enriched with graphite flakes used in ambient with room temperature was studied in [12]. These results showed especially the importance of the choice of melt entry during their fabrication but did not delve deeper into the heat sinks’ efficiency if conditions would be any different from the room temperature and if natural convection would be limited due to use in enclosed space as is often the case in real applications.

As polymer heat sinks that have naturally high emissivity heat dissipation via radiation cannot be ignored, the heat sinks’ design should be subjected to this mode of heat transfer. Importance of radiation on the overall cooling efficiency was studied in [13,14,15,16]. Huang, L. et al. in [14] state that comparing high emissivity coating with a low emissivity one applied to an LED filament bulb can reduce its temperature by 10%. Bravo, R.H. et al. in [16] showed in their publication that in case of an array of electronic chips mounted between two parallel plates the thermal radiation represented 33% of the total heat transfer.

This paper shows how heat dissipation from a polymer heat sink changes when subjected to different ambient temperatures. Change of air properties with rising temperature by tens of degrees is minimal; therefore, the heat transfer coefficient by convection stays almost the same. Hence, using a metal heat sink with glossy surface in an ambient where the temperature should rise would lead to rise of the entire cooling system by the same margin. However, if a heat sink with high surface emissivity is applied, this increase in ambient temperature can lead to significant rise of heat dissipation via radiation, therefore better cooling efficiency.

## 2. Materials and Methods

### 2.1. Geometry of Tested Heat Sinks

**Figure 1:**
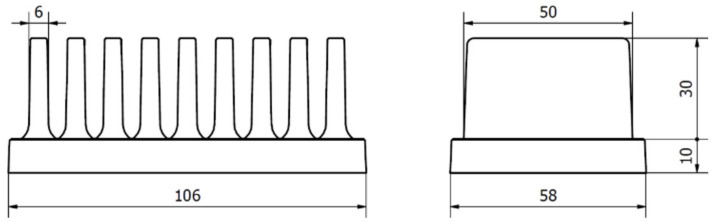
Geometry of tested heat sinks (dimensions are in mm).

The tested heat sinks had dimensions (58 × 40 × 106) mm with 9 fins (details are shown in Figure 1). For the study’s purpose the chosen design was simplistic to allow an easy way to calculate dissipated heat through the heat sinks’ fins according to [17]. Heat sinks were produced from Polyamide 66 as the matrix material with graphite flakes as the main filler that ensured the high thermal conductivity. This blend is commercially available and was produced by Avient (Prague, Czech Republic). The thermal conductivity of this material ranged from 5 to 20 W/m·K depending on the filler orientation, density 1640 kg/m^3^. The heat capacity is unknown (not essential because the data presented in this paper is for the thermal steady state). All the values were taken from the official data sheet.

**Figure 2:**
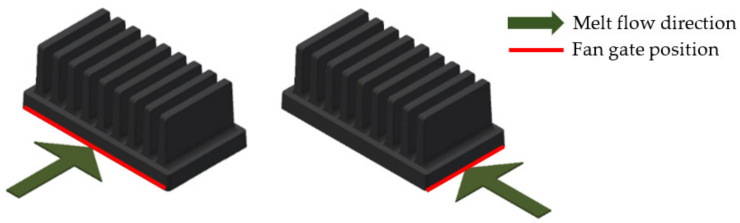
Melt entrance along sinks’ edge (red), direction of melt flow depicted by green arrow. Left—edge of longer base’s side (melt flow parallel to the fins), right—edge of shorter base’s side (melt flow perpendicular to the fins).

To compare the influence of the melt flow, two polymer heat sinks of the same geometry were used in the measurement. The difference between the two heat sinks was in the melt entry point during production. One heat sink was produced with the melt entry along the heat sink’s longer base’s edge so the melt would flow in parallel with the heat sink’s fins and the other had the melt entry on the shorter base’s edge perpendicular to the heat sink’s fins. Entry points of both measured heat sinks are shown in Figure 2.

**Figure 3:**
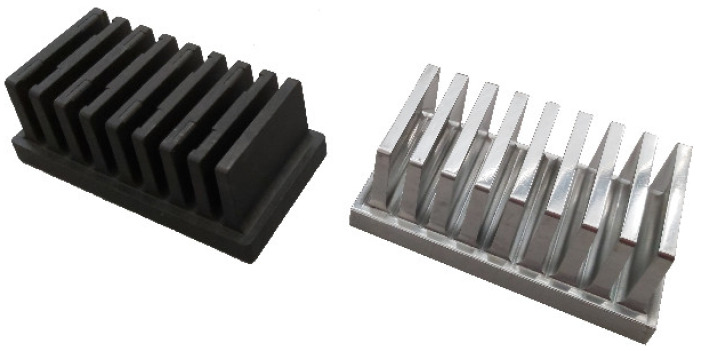
Measured polymer (left) and aluminum (right) heat sinks side by side.

Furthermore, an aluminum heat sink of the same geometry was also compared with the two polymer heat sinks. The aluminum heat sink had thermal conductivity 130 W/m·K and a low surface emissivity. One of the measured polymer heat sinks with high emissivity and the aluminum heat sink with low emissivity are shown in Figure 3.

The results from the cooling efficiency were used to demonstrate the importance of the surface emissivity in application with higher ambient temperatures.

### 2.2. Measurement Description

**Figure 4:**
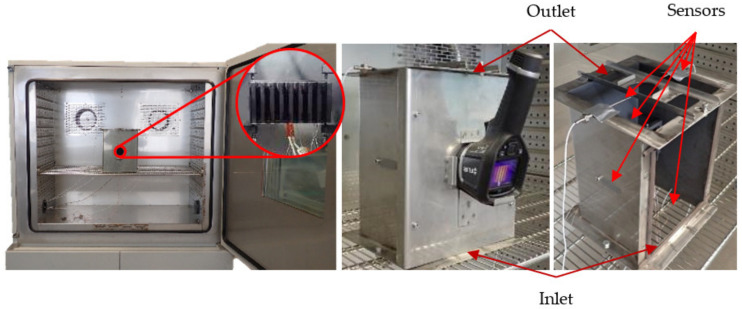
Heat sink placement inside the measurement box (left), measurement box inside the thermostatic chamber with attached thermal imaging camera (middle), detail of the measurement box (right).

The measured polymer heat sinks were situated inside a measurement box, which was placed inside a thermostatic chamber Binder MK 720 (manufacture Binder, Hradec Kralove, Czech Republic) during the conducted measurements. The box with one of the measured heat sinks inside the thermostatic chamber is shown in Figure 4. The box’s holders touched the heat sinks only in four points to ensure minimal heat conduction away from the heat sinks and into the box itself. The thermostatic chamber served as the source of the desired ambient temperature and the box protected the heat sinks from any unwanted forced convection created by the chamber’s fans. The box’s front door also served as a placement of the FLIR E5 thermographic camera.

**Figure 5:**
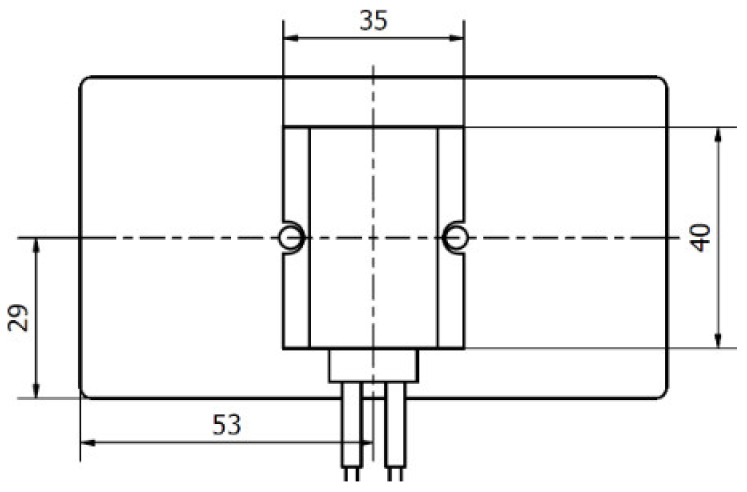
Heater placed in the middle of the heat sink’s base (dimensions are in mm).

The heat leading to the heat sink was generated with a resistance heater. The contact surface area between the heat sink and the heater was 35 × 40 mm. The placement of the heater on the heat sink’s base is shown in Figure 5. A thermally conductive paste with a thermal conductivity of 4.2 W/m·K was applied between the heat source and the heat sink base. This paste ensured a good thermal contact, which was the same for every experiment.

The heat output of the heater was set to 15 W with a precision of ±0.1 W. It was further controlled with a LabView program to ensure the same amount of heat was generated regardless of the heater’s resistance change caused by increase of its temperature. Every measurement was repeated with detaching and reattaching the heater. The measurements’ results were compared to eliminate the possibility of the bad contact between the heater and the heat sink leading to the measurement error.

**Figure 6:**
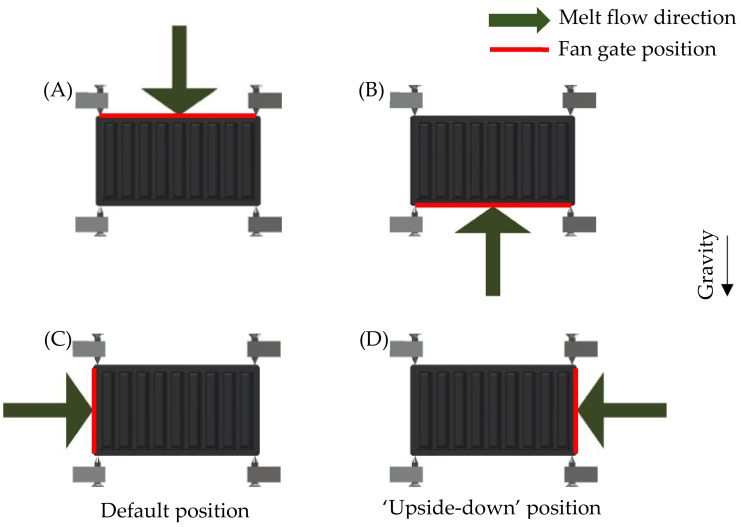
Orientation of the melt entry for both measured heat sinks in two different measurement positions, left—default position, right—‘upside-down’. Melt entrance along the red edge, melt flow in the direction of the green arrow. (**A**) heat sink with melt entrance along the longer base’s edge (parallel to fins)—default position, (**B**) heat sink with melt entrance along the longer base’s edge (parallel to fins)—upside-down position, (**C**) heat sink with melt entrance along the shorter base’s edge (perpendicular to fins)—default position, (**D**) heat sink with melt entrance along the shorter base’s edge (perpendicular to fins)—upside-down position.

Both measured polymer heat sinks were measured in two positions as in [12]. The positions were named ‘default’ and ‘upside-down’. This led to change of the filler orientation in relation to gravity and therefore buoyancy in case of the polymer heat sink with the melt entry point parallel to the fins, which as was shown in [12] led to different cooling efficiencies. Change of the melt flow direction in regard to gravity is shown in Figure 6.

## 3. Measurement Uncertainty Analysis

To ensure maximal measurement precision and to minimize all the possible errors the following measures were taken.

The used thermocouple was calibrated for different ambient temperatures beyond its standard measurement precision of ±2.2 °C. Its new maximum deviation was set to only ±0.2 °C.

**Figure 7:**
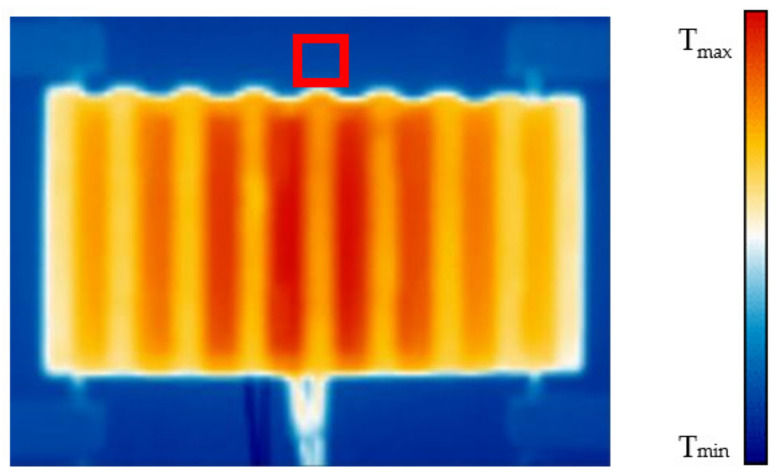
Thermogram with an indicated area where the thermocouple for the verification of the correct measured temperature via thermal imagery was placed.

When a steady state of the measurements was achieved a thermal camera FLIR E5 with measurement precision of ±2 °C and with resolution of 120 × 90 pixels was used to further analyze the temperature field on the heat sinks’ fins. To further increase its precision another type K thermocouple calibrated the same way as the previous one was placed in the back wall inside an area which was seen in all the measurements. As this wall was dyed with a graphite spray of known emissivity the thermal imagery results could be further adjusted by comparing its values inside the area where the thermocouple was situated. This area is shown in Figure 7.

To ensure the ambient temperature was as close to its desired value, two resistance thermometers PT100 were placed at the bottom of the measurement box which served as the inlet once the air flow induced by the natural convection was stabilized. These thermometers measured with the measurement error of ±0.2 °C.

## 4. Results

All the configurations were measured multiple times (as described in Section 2.2) with good repeatability. The data presented was generated by averaging the results for each measured configuration.

### 4.1. Temperature Comparison

Both compared polymer heat sinks were measured with 15 W thermal input power of the heater in two positions as described in Section 2.2. The ambient temperature was maintained at 50 °C. The temperatures of the heater are shown in Table 1.

These results can be compared with the results from measurement for ambient temperature 20 °C. These results are shown in Table 2.

By comparing these results, it can be concluded that with higher ambient temperature the importance of the melt entry point into the mold diminishes and the same stands for the importance of the heat sink’s position if the melt entry point is parallel to fins. Going from ambient temperature 20 °C to 50 °C decreased the heater’s temperature between the best and the worst heat sinks’ performances by 49.3% from 6.7 °C to only 3.4 °C. In case of efficiency change for the heat sink with melt entering along the base’s longer edge the heater’s temperature difference dropped by 25% from 2.8 °C to 1.9 °C.

### 4.2. Convection-Radiation Heat Transfer Comparison

From the previously shown results it is visible that the difference between cooling efficiencies of the measured heat sinks is significantly lower for the ambient temperature 50 °C compared to the results for the ambient temperature 20 °C. This phenomenon shows that the means of heat dissipation from the heat sinks is significantly different. The ratio between the dissipation via convection and radiation must change if the heat sinks have an overall higher temperature as the radiation heat transfer rises with the fourth power of the surface’s absolute temperature.

To prove this change in heat transfer means from the heat sinks to the ambient the same approach to the efficiency comparison as in [12] was used. The equations used in this approach are to be found in [17] and it analyses the amount of dissipated heat via heat sink’s fins.

Firstly, the temperature field on the heat sinks’ fins is measured with thermal imagery and the temperature difference between the heat sinks’ fins temperature and ambient is calculated.

(1) $$\Delta T_{ij}=T_{fin,ij}-T_{ambient}.$$

The index i refers to the fin number and j to the surface $S_{j}$. To calculate heat transfer by convection Rayleigh number is determined.

(2) $$Ra_{ij}=\frac{g\cdot \beta_{air}\cdot \Delta T_{ij}\cdot L_{j}^{3}}{\nu_{air}\cdot \alpha_{air}},$$

where g is gravitational acceleration, $\beta_{air}$ is thermal expansion coefficient of the ambient air, $\nu_{air}$ is kinematic viscosity of the ambient air, $\alpha_{air}$ is thermal diffusivity of the ambient air and $L_{j}$ is characteristic dimension of the surface $S_{j}$ ($L_{1},L_{2},L_{3}$—fin length, $L_{4},L_{5}$—surface area divided by its perimeter). The air properties used for the calculation are determined from so called film temperature on the fins.

(3) $$T_{film,ij}=\frac{T_{fin,ij}+T_{ambient}}{2}.$$

Next, convective heat transfer is calculated as a function of Rayleigh number, ambient air’s thermal conductivity and characteristic dimension.

(4) $$h_{conv,ij}=f_{j}\left(Ra_{ij},\;L_{j},k_{air}\right),$$

where $k_{air}$ is ambient air’s thermal conductivity at the film temperature. The radiative heat transfer was calculated using the known surface emissivity and Stefan–Boltzmann constant.

(5) $$h_{rad,ij}=\varepsilon \sigma \left(T_{fin,ij}+T_{ambient}\right)\cdot \left(T_{fin,ij}^{2}+T_{ambient}^{2}\right),$$

where the surface emissivity ε was set to 0.95 and σ is Stefan–Boltzmann constant.

**Figure 8:**
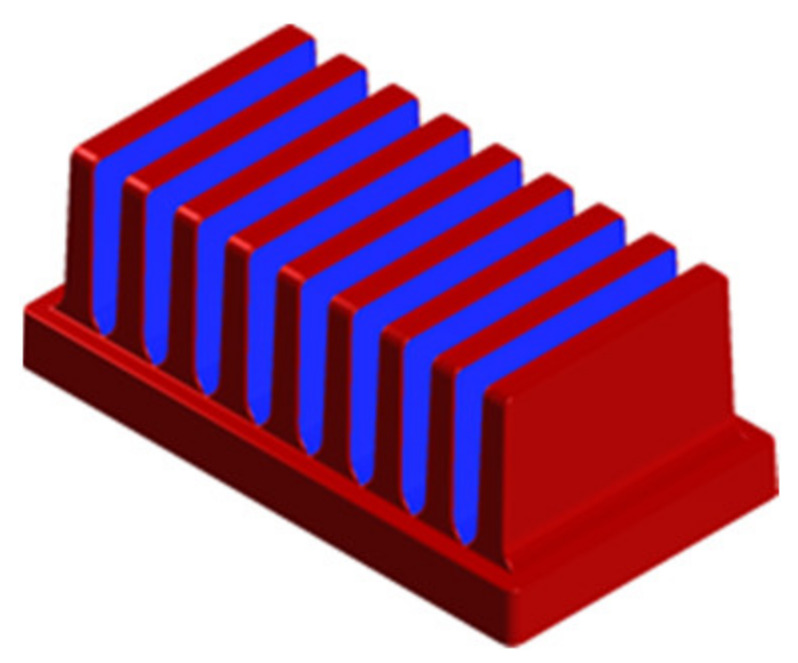
Heat sinks’ surfaces without taking view factor into account (red) and with taking view factor into account (blue).

For the fins’ surface that radiated heat between themselves a view factor was calculated. Figure 8 shows the surfaces where the view factor was considered (blue) and where it was not (red). The outer surfaces without taking view factor into account formed together an area of 18,963 mm^2^ and the internal surfaces where the view factor was considered created together an area of 23,331 mm^2^, which is 55.2% of the entire surface. This means that less than a half of the heat sink’s surface can radiate heat away freely.

The final individual heat flows from fins $Q_{i}$ were calculated:

(6) $$Q_{i}=\underset{j}{\sum }Q_{ij},\;\;\;\;\;\;\;Q_{ij}=\left(h_{conv,ij}+h_{rad,ij}^{*}\right)\cdot A_{j}\cdot \Delta T_{ij},$$

where h is heat transfer coefficient, the index i refers to the fin number and j refers to individual surface areas of fins $A_{j}$.

Table 3 shows calculated heat flows from fins of the heat sink made with the melt flow parallel to its fins for the measured heat sinks in both 20 °C and 50 °C ambient temperatures and differences between them.

Additionally, Table 4 shows calculated heat flows from fins of the heat sink made with the melt flow perpendicular to its fins for the measured heat sinks in both 20 °C and 50 °C ambient temperatures and differences between them.

From these results it is obvious that heat transfer by radiation grows significantly with higher ambient temperature. As the same amount of power is generated inside the heater and the radiative heat transfer grows, the heat transfer via convection must drop. Calculated heat transfer from heat sinks’ fins also shows drop in the overall heat transfer and especially for the case of the heat sink produced with melt flow perpendicular to its fins. As the heat sink’s base is the warmest part, the amount of heat radiated away to the ambient rises here the most, hence the total amount of heat transferred away from fins drops.

### 4.3. Convection-Radiation Heat Transfer Comparison

The same type of measurement was done for an aluminum heat sink of the same geometry. The heater was attached to the aluminum heat sink with input power of 15 W and the source’s temperature was measured in both conditions with ambient temperature 20 °C and 50 °C. Table 5 shows how the heater‘s temperature changed between the two measurement conditions for the aluminum heat sink and both polymer heat sinks.

In case of the aluminum heat sink, the heater’s temperature rose by 29.9 °C with the ambient temperature change of 30 °C. In case of the polymer heat sink produced with the melt flow parallel to its fins and positioned in the most suitable position to induced natural convection the heater’s temperature increment for the same ambient temperature change was 27.6 °C. This proves that the amount of heat radiated away thanks to its high emissivity rises with increasing the ambient temperature. This phenomenon is especially visible in case of the heat sink produced with melt flow perpendicular to its fins where the heater’s temperature rose only by 24.2 °C. This is caused by heat sink’s unideal heat conductivity to its fins; therefore, its overheated base radiates away even higher amount of heat.

**Figure 9:**
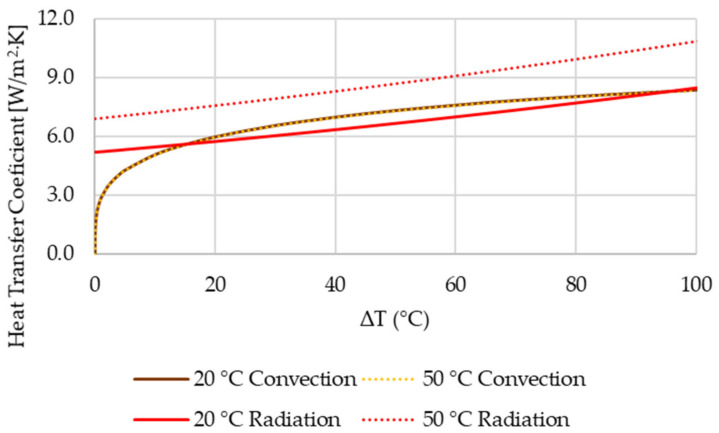
Comparison of convective and radiative heat transfer coefficient dependent on temperature difference between the heat sink surface and ambient surfaces for ambient temperatures of 20 and 50 °C.

The heat transfer by convection changes minimally if the ambient air temperature should be changed only by tens of degrees. Change in radiative and convective heat transfer coefficient are shown in Figure 9. The convective heat transfer coefficient was calculated for fins’ length of 50 mm and the radiative heat transfer was calculated for emissivity of 0.95.

**Figure 10:**
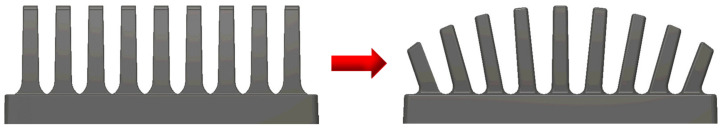
Possible heat sink design change to allow better heat dissipation by radiation.

In case of high ambient temperature heat sinks with high emissivity have the radiative heat transfer coefficient always higher than the conductive one (see in Figure 9). Traditional heat sink geometries are designed with having especially good spacing between fins in mind to allow the best heat dissipation via convection. However, in cases where heat dissipation via radiation should be dominant it is important to change the standard design to allow more heat to be radiated to the ambient. This can be achieved by not having fins placed parallelly to each other and also with variable length as shown in Figure 10. This way the view factor rises and more heat can be radiated away to the ambient.

## 5. Conclusions

Change of the heat transfer ratio from the polymer heat sink to the ambient was shown. It was proved that with higher ambient temperature the heat transfer via radiation gains importance. It can even dominate over heat transfer via conduction therefore thermally conductive polymers show their functionality especially in non-ideal conditions with high ambient temperature.

The aluminum heat sink of the same geometry as the measured polymer ones showed increase of 29.9 °C with the ambient temperature change from 20 to 50 °C. This shows that the change of the heater temperature on the low emissivity aluminum heat sink replicates the shift of ambient temperature. This increase was only 24.2 °C in the case of the polymer heat sink that was made with the melt flow perpendicular to its fins as the radiative heat transfer especially from its base rose drastically.

Being used in ambient temperatures higher than room temperature allows the polymer heat sinks with natural high surface emissivity to dissipate heat via radiation more easily than in conditions with low ambient temperature. This is due to a higher radiative heat transfer coefficient as it rises with fourth power of absolute temperature and also the overall higher temperature of the heat sink.

Natural high surface emissivity given to polymers without any additional coating therefore any additional manufacturing processes leads to further cost reduction in comparison with metal heat sinks if similar emissivity would need to be achieved.

To further increase the heat dissipation by radiation, it is necessary to modify the shape and position of the fins and their distribution to increase the area from which the heat is radiated to the surroundings, as shown in Figure 10.

It has also been proven that the importance of the melt entry point during the production for the overall heat sink efficiency as shown in [12] slowly loses its importance in case of higher ambient temperatures. This is because the heat dissipation via convection diminishes and the heat dissipation via radiation rises. Additionally, the heat sinks’ positioning loses its importance as the effectivity of the heat transfer by radiation is not dependent on the heat sink’s spatial orientation.

## Figures and Tables

**Table 1:** Heater temperature in all polymer heat sinks conducted measurements for ambient temperature 50 °C. The letters in parentheses refer to positions according to Figure 6.

	Default Position (°C)	Upside-Down (°C)	Position Difference (°C)
Melt entrance along the longer base’s edge (parallel to fins)	99.0 (A)	97.1 (B)	1.9 (A)-(B)
Melt entrance along the shorter base’s edge (perpendicular to fins)	100.3 (C)	100.5 (D)	0.2 (D)-(C)
Melt Entry Difference (°C)	1.5 (C)-(A)	3.4 (D)-(B)	

**Table 2:** Heater temperature in all polymer heat sinks conducted measurements for ambient temperature 20 °C [12]. The letters in parentheses refer to positions according to Figure 6.

	Default Position (°C)	Upside-Down (°C)	Position Difference (°C)
Melt entrance along the longer base’s edge (parallel to fins)	72.3 (A)	69.5 (B)	2.8 (A)-(B)
Melt entrance along the shorter base’s edge (perpendicular to fins)	76.1 (C)	76.2 (D)	0.1 (D)-(C)
Melt Entry Difference (°C)	3.9 (C)-(A)	6.7 (D)-(B)	

**Table 3:** Calculated heat transfers from fins of the polymer heat sink with melt flow parallel to its fins for both 20 °C and 50 °C ambient temperatures. The letters in parentheses refer to positions according to Figure 6.

Heat Sink	Melt Entering Parallelly to Fins (Default Position) (A)		Melt Entering Parallelly to Fins (Upside-Down Position) (B)	
Ambient temperature (°C)	20	50	20	50
Convective heat flow (W)	7.7	6.8	7.6	6.9
Radiative heat flow (W)	3.2	3.8	3.1	3.9
Total heat flow (W)	10.9	10.5	10.8	10.7

**Table 4:** Calculated heat transfers from fins of the polymer heat sink with melt flow perpendicular to its fins for both 20 °C and 50 °C ambient temperatures. The letters in parentheses refer to positions according to Figure 6.

Heat Sink	Melt Entering Perpendicularly to Fins (Default Position) (C)		Melt Entering Perpendicularly to Fins (Upside-Down Position) (D)	
Ambient temperature (°C)	20	50	20	50
Convective heat flow (W)	7.2	6.1	7.2	6.0
Radiative heat flow (W)	2.9	3.4	3.0	3.4
Total heat flow (W)	10.1	9.5	10.2	9.4

**Table 5:** Heater‘s temperature change with change of the ambient temperature on different measured heat sinks. The letters in parentheses refer to positions according to Figure 6. The data for the ambient temperature 20 °C are from [12].

Heat Sink	Heater Temperature for Ambient Temperature 20 °C (°C)	Heater Temperature for Ambient Temperature 50 °C (°C)	Difference (°C)
Aluminum	65.7	95.6	29.9
Polymer made with melt flow along fins (B)	69.5	97.1	27.6
Polymer made with melt flow perpendicular to fins (D)	76.1	100.3	24.2

## Data Availability

Not applicable.
